# Supplementary material for: The interactome of intact mitochondria by cross-linking mass spectrometry provides evidence for coexisting respiratory supercomplexes
Source: Mol Cell Proteomics. 2017 Dec 8;17(2):216–32. doi: 10.1074/mcp.RA117.000470 (PMC5795388; doi:10.1074/mcp.RA117.000470)
Supplement: Supplemental Data [file supp_RA117.000470_133922_0_supp_23978_fzffwf.zip › spectra_annotation/mito_DR_spectra_annotation/241-1-6-1-6-1.pdf]

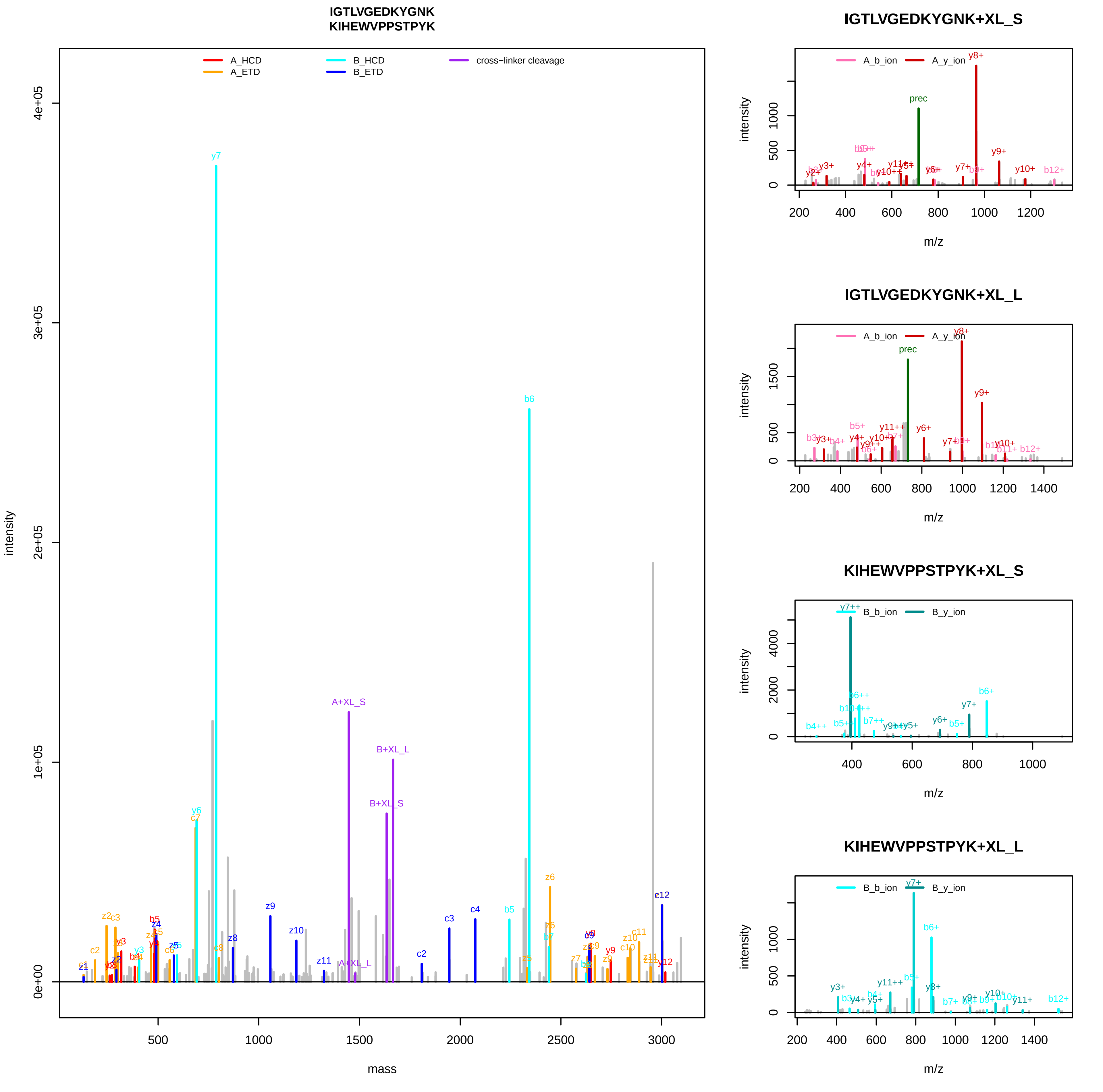
IGTLVGEDKYGNK
KIHEWVPPSTPYK
A_HCD
A_ETD
B_HCD
B_ETD
cross−linker cleavage
intensity
mass
0e+00
1e+05
2e+05
3e+05
4e+05
500
1000
1500
2000
2500
3000
y7
b6
A+XL_S
B+XL_L
B+XL_S
A+XL_L
y6
c7
z6
c12
z9
z10
z11
c2
c3
c4
b5
z8
c8
b5
z4
c11
c10
z10
y9
y12
IGTLVGEDKYGNK+XL_S
A_b_ion
A_y_ion
prec
y8+
y9+
y10+
y7+
y3+
y4+
b12+
m/z
IGTLVGEDKYGNK+XL_L
y11++
y6+
b5+
KIHEWVPPSTPYK+XL_S
B_b_ion
B_y_ion
y7++
b6+
y7+
y6+
b6++
b10++
b7++
b5+
KIHEWVPPSTPYK+XL_L
y7+
b6+
y8+
y11++
b5+
b12+
y10+
y11+
y3+
